# Radiological diagnosis of the inner ear malformations in children with sensorineural hearing loss

**DOI:** 10.1259/bjro.20180050

**Published:** 2019-06-14

**Authors:** Bernadine Quirk, Adam Youssef, Mario Ganau, Felice D'Arco

**Affiliations:** 1 Great Ormond Street Hospital for Children NHS Foundation Trust,; 2 Department of Neurosurgery, Oxford University Hospitals NHS Foundation Trust,

## Abstract

Malformations in either the inner ear, vestibulocochlear nerve (VIIIth) or auditory cortex of the brain can lead to congenital sensorineural hearing loss (SNHL). In most cases the underlying disorders involve the membranous labyrinth at a microscopic level and therefore radiological examinations are entirely normal. In a significant proportion however (up to 20%), there are abnormalities visualized in the inner ear and/or the VIIIth nerve; the type of abnormality is relevant for the surgical planning of a cochlear implant. Imaging and the accurate radiological identification of the affected inner ear structures therefore plays an integral role in the clinical evaluation of sensorineural hearing loss. In this pictorial review, we describe the main malformations of the inner ear in view of recent classifications and briefly explore the surgical implications.

## Introduction

Malformations in either the inner ear, vestibulocochlear nerve (VIIIth) or auditory cortex of the brain can lead to congenital sensorineural hearing loss (SNHL).^1^ In most cases, the underlying disorders involve the membranous labyrinth at a microscopic level and therefore radiological examinations are entirely normal. In a significant proportion however (up to 20%),^2^ there are abnormalities visualized in the inner ear and/or the VIIIth nerve^3^; the type of abnormality is relevant for the surgical planning of a cochlear implant. Imaging and the accurate radiological identification of the affected inner ear structures therefore plays an integral role in the clinical evaluation of SNHL. In this pictorial review, we describe the main malformations of the inner ear in view of recent classifications and briefly explore the surgical implications.

## The role of imaging

MRI and CT are complementary in the pre-operative work-up.^4^ CT enables accurate anatomical surgical planning; visualizing the bony structures of the inner, middle and external ear and anatomical variants that may influence surgery. MRI is critical in the assessment of the cochlear nerve, in addition to identifying the labyrinthine portion of the inner ear and any associated brain anomalies.^5,6^

Newer cone beam techniques offer superior imaging resolution but at lower radiation doses compared to traditional multidetector CT; an obvious advantage in the paediatric setting and in the presence of very small anatomical structures such as the ossicles. The otic capsule and inner ear structures are fully formed at birth and remain stable in size over time. A recent review^7^ summarizes the usefulness of normative values in identifying and classifying anomalies, rather than relying on visual inspection alone. This is particularly relevant in the diagnosis of different subtypes of cochlear hypoplasia’s, where the identification of subtle abnormalities can be challenging, and small cochleae may be overlooked.

High resolution, three-dimensional *T*_*2*_W (*T*_2 _weighted) images on MRI (with a spatial resolution approaching 0.4 mm) enables visualization of tiny cochlear structures such as the interscalar septum and lamina spiralis (since minor malformations with subtle osseous abnormalities are not always visualized on CT). As expected, neural structures are well delineated on MRI, namely the facial (VIIth), vestibular and cochlear branches of the VIIIth cranial nerves. Depiction of the latter is of paramount importance as although similar appearing anomalies have comparable therapeutic options, it is the integrity of the cochlear nerve which ultimately governs functional outcome.^8^

## Normal anatomy

**Figure 1.**
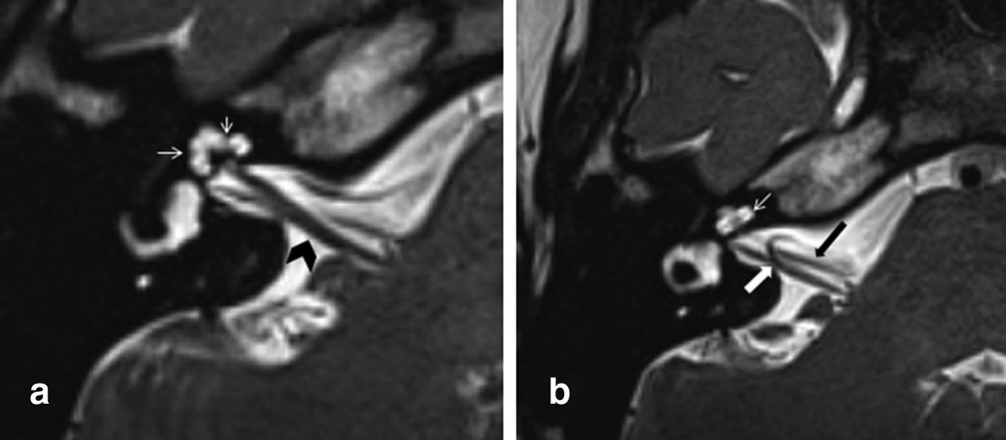
Axial high-resolution 3D *T*_2_WI of the labyrinth showing a normal cochlea with well-formed interscalar septum dividing the different cochlear turns (arrows in A) and the lamina spiralis dividing scala vestibuli and scala tympani (thin arrow in B). The modiolus is visible in A as a hypointense triangular area. The VIIIth nerve and its cochlear and vestibular divisions are noted (arrowhead in A). The VIIIth (white thick arrow) and VIIth (thick black arrow) nerves in the cisternal portion are seen in B (slightly cranial to A). 3D, three-dimensional; *T*_2 _WI, *T*_2 _weighted imaging.

The inner ear occupies the petrous part of the temporal bone and is made up of membranous and osseous labyrinths. The osseous labyrinth is contained within the otic capsule which is the densest structure in the body. It consists of the cochlea, vestibule and three semi-circular canals (SCCs), along with the vestibular and cochlear aqueducts. The MRI anatomy of the otic capsule and labyrinth are demonstrated in Figure 1.

The vestibule is the central portion of the osseous labyrinth, which is continuous anteroinferiorly with the cochlea and the vestibular aqueduct (VA) posteriorly.^1^ The three SCCs arise from its lateral, posterior and superior margins respectively and the superior SCC joins the posterior SCC at a common segment called the common crus. The junction of the common crus and the vestibule is where the VA arises before travelling along the postero inferior petrous bone. It contains the membranous endolymphatic duct and sac and should be no larger than 1 mm at its midpoint and 2 mm at the opercular opening^7,9^ or (according to other studies) should be no larger than the adjacent ascending limb of the posterior SCC.^10^

The cochlea (from the Greek "kokhlias" meaning snail) is the part of the inner ear responsible for hearing and is a partitioned structure that spirals approximately 2.25 to 2.75 times around a central perforated bone known as the modiolus.^11^ As it spirals superiorly from the base to the apex its diameter gets progressively smaller. Inside, an osseous spiral lamina which projects laterally from the modiolus, partitions the cochlear spiral into the anteriorly located scala vestibuli and the more posterior scala tympani, connected via the helicotrema opening at the cochlear apex.^1^ They both contain perilymph which is of CSF density and it is the scala tympani which is the surgical target for cochlear implantation.^12^ The scala media (or cochlear duct) is interposed between the other scalae and is beyond the resolution of current imaging. It contains the endolymph and is an integral part of the membranous labyrinth. The cochlear aqueduct runs inferiorly and roughly parallel to the internal auditory canal (IAC) and carries peri-lymph from the basal turn of cochlea to the posterior fossa.

**Figure 2.**
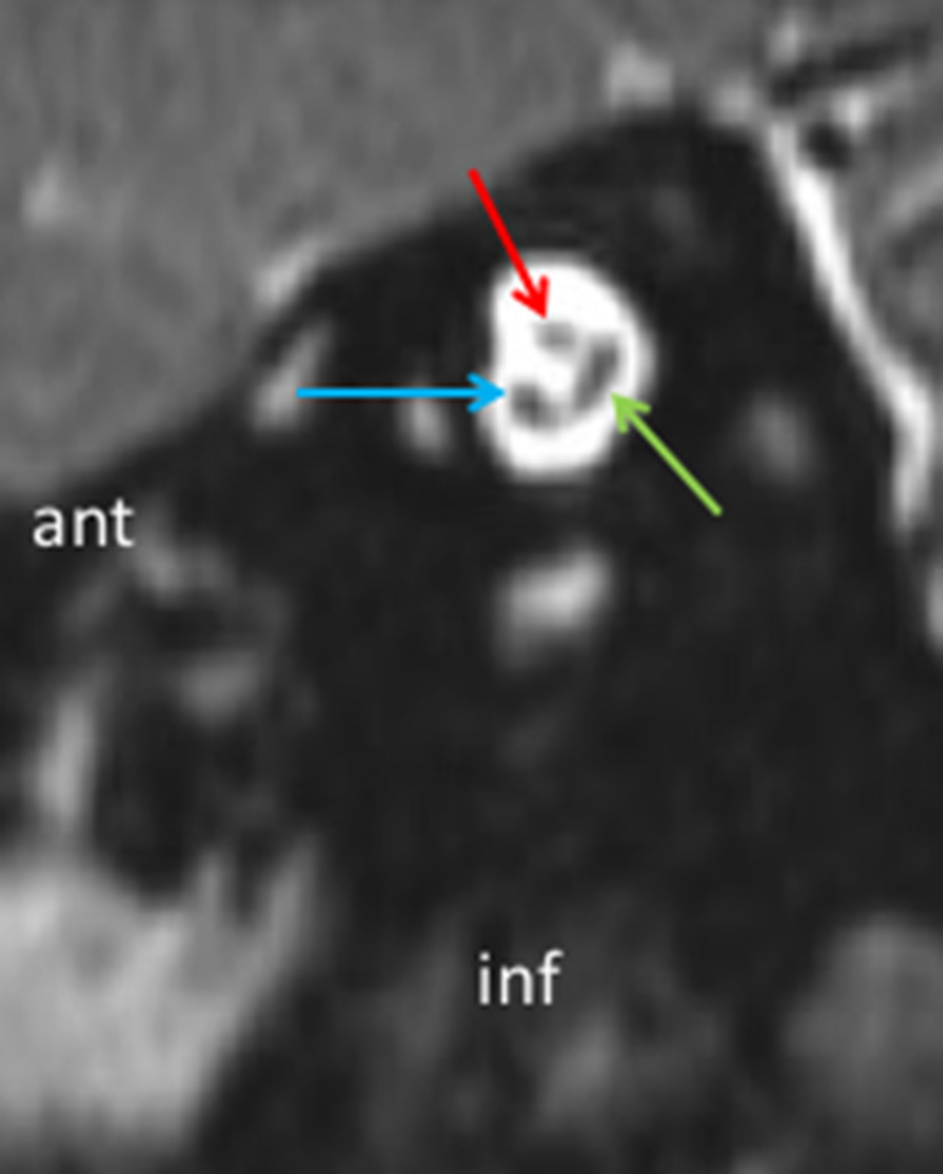
Parasagittal MRI reformat perpendicular to the IAC showing VIIth nerve (red arrow), cochlear nerve (blue arrow) and vestibular nerves (green arrow) with the inferior and superior vestibular branches still unified. IAC, internal auditory canal.

The IAC is separated from the vestibule by the lamina cribrosa and contains the cochlear aperture in its fundus, through which the cochlear nerve enters the cochlea. The four nerves running in the IAC are nicely visualized on MR sagittal oblique views; the facial nerve lies anterosuperiorly, the cochlear nerve anteroinferiorly, the superior vestibular nerve posterosuperiorly and the inferior vestibular nerve posteroinferiorly (Figure 2). The four nerves should be of a similar diameter and signal intensity in parasagittal images acquired perpendicular to the IAC long diameter. The facial nerve, in its cisternal segment, is approximately half the size of the vestibulocochlear nerve in the axial plane. The cisternal and canicular segments of the cochlear nerve are CSF filled and therefore the preferred modality for characterization of any cochlear nerve anomalies is a high resolution, heavily weighted *T*_2_W MRI.

## Inner ear malformations

**Figure 3.**
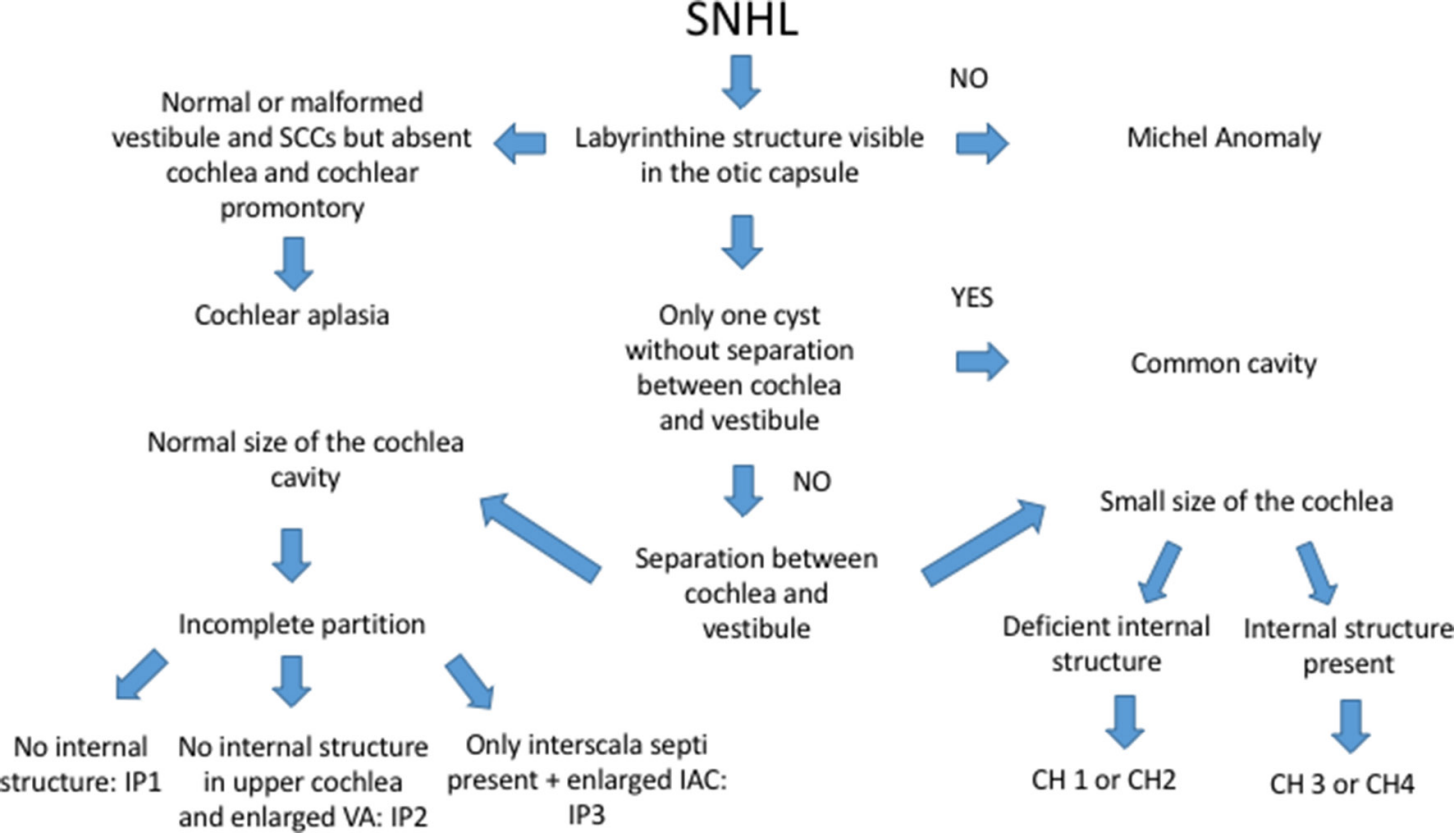
Chart for evaluation of SNHL in children. SNHL, sensorineural hearing loss.

A wide spectrum of malformations have been described; ranging from complete inner ear aplasia to relatively milder anatomical variants (Table 1: Radiological features of SNHL classification in children and Figure 3: Chart for evaluation of SNHL in children). Abnormalities are proposed to arise due to developmental arrests in the normal inner ear embryogenesis at various time points^13,14^; as a general rule the earlier the arrest, the more severe the dysplasia. Interestingly, recent histopathological studies have shown a more complex etiopathogenesis than the time of insult only, with differentiation between various genetic and toxic causes.^3^

**Table 1.** Radiological features of SNHL classification in children

**DIAGNOSIS**	**Cochlea**	**Vestibule**	**IAC**	**Semi-circular canal**	**Facial nerve canal**
CLA	Absent	Absent	Aplasia/ hypoplasia	Absent	Aberrant
Rudimentary Otocyst	Absent	Absent	Absent (usually)	Tiny parts may be present	Absent
CA with normal labyrinth	Absent	Normal	Hypoplasia	Normal	Aberrant
CADV	Absent	Variably dilated	Hypoplasia	Variably dilated	Aberrant
CC	Rudimentary	Rudimentary	Usually enters at its centre	Often horizontal SCC	Aberrant
IP-1	Normal size, cystic outline, no modiolus/ISS	Usually enlarged	Enlarged	Often horizontal SCC	Normal
IP-2	Normal size, cystic apex, normal modiolus/ISS	Minimally dilated	Normal	Normal	Normal
IP-3	Normal size, no modiolus, ISS present but dysplastic	Normal	Bulbous, direct cochlear connection	Normal	Aberrant
CH-1	Small, bud-like, no ISS/modiolus	Normal	Direct cochlear connection	Normal	Normal
CH-2	Small, normal outline, defective ISS/modiolus	Normal	Direct cochlear connection	Normal	Normal
CH-3	Small (<2 turns), the “unwound cochlea” in BOR, small modiolus, short ISS	Hypoplasia	Normal	Hypoplasia	Normal
CH-4	Small, hypoplastic apical/middle turns	Normal	Normal	Normal	Aberrant
Dysplastic SCC	Normal/but can be varyingly abnormal	Normal	Normal	Dysplastic	Normal
Cochlear nerve aplasia/hypoplasia Type 1	Normal	Normal	Stenotic/ atretic	Normal	Normal
Cochlear nerve aplasia/hypoplasia Type 2a	Normal	Normal	Stenotic/ atretic	Normal	Normal
Cochlear nerve aplasia/hypoplasia Type 2b	Normal	Normal	Normal	Normal	Normal

BOR, Branchio-Oto-Renal; CADV, CA with a dilated vestibule; IAC, internal auditory canal; ISS, interscalar septa; SNHL, sensorineural hearing loss.

### Complete labyrinthine aplasia (CLA)

**Figure 4.**
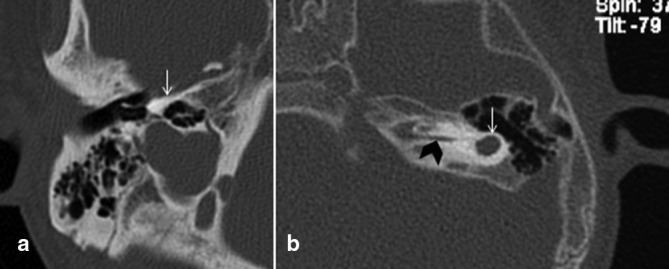
Axial CT showing complete labyrinthine aplasia in patient with LAMM syndrome (Congenital deafness with labyrinthine aplasia, microtia, and microdontia), no labyrinthine is seen in a small and dense otic capsule (arrow). Figure 4B Rudimentary otocyst (white arrow) with narrowed IAC (black arrowhead). IAC, internal auditory canal.

CLA (or the Michel deformity) is defined by a complete absence of the inner ear structures and was first described by Michel in 1863.^15^ It is the most severe inner ear abnormality and extremely rare; comprising less than 1% of all cochlear bony malformations.^16,17^ The proposed pathophysiology is an arrest (acquired or genetic) in the otic placode development at or before week 3^18^; i.e. before the formation of the otic capsule and membranous labyrinth (Figure 4A). On imaging, there is bony atresia or narrowing of the IAC with absence of a detectable VIIIth nerve on MRI and therefore cochlear implantation is contraindicated.^1^ It is mostly bilateral, but may be unilateral and, in such cases where there is CLA on one side there is frequently contralateral severe dysplasia.^1^ A plethora of associated otic capsule anomalies are often observed and a case series by Ozgen and Sennaroğlu^16^ describes typical findings such as absent round and oval windows, and an anomalous course of the facial nerve.^16^ The middle ear may also be abnormal with a dysplastic stapes and variable hypoplasia of the middle ear cavity and petrous bone.^16^ Of note, complete absence of labyrinth is seen in both CLA and labyrinthitis ossificans, however it is the flattening of the middle ear medial wall (as there is no cochlea to form the cochlear promontory), characteristic of CLA, that helps differentiate the two entities together with the clinical context.^16,17^ Beyond the petrous bone, anomalies in the posterior fossa, skull base and craniocervical junction are often also seen and include platybasia and jugular venous and sinus variants.^1^

### Rudimentary otocyst

In this severe deformity there is a tiny (mms) ovoid or round cavity within the otic capsule and the IAC is usually absent ^3^ (Figure 4B), although occasionally there may be rudimentary IAC formation. If the IAC is absent this implies that there is no communication with the brainstem,^8^ and as such functionally it is very similar to CLA. The embryological insult is therefore thought to be early; around the third week.^3^

### Cochlear aplasia (CA)

**Figure 5.**
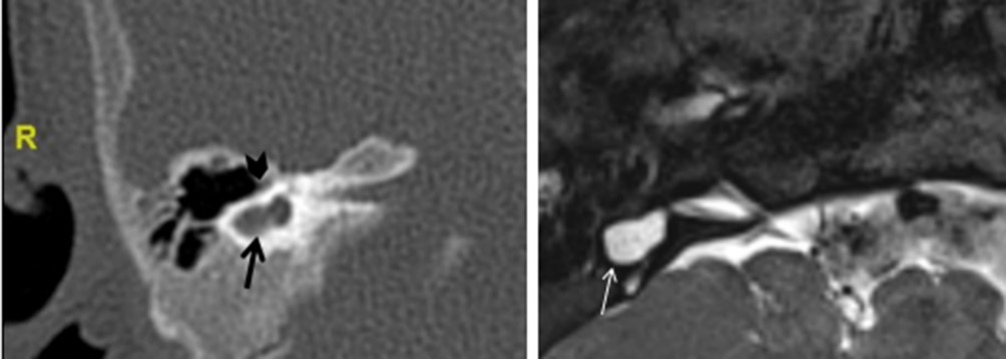
Cochlear aplasia on CT (left) and MRI (right) on axial planes. Note dysmorphic vestibule (arrows) and absent cochlear promontory (arrowhead) in keeping with cochlear aplasia with a dilated vestibule.

CA describes the complete absence of the cochlea and is again rare (3% of all cochlear anomalies).^1,18^ Dense sclerosis and an anteriorly dislocated labyrinthine segment of the facial (VIIth) nerve are shown on CT in lieu of a normal cochlea (Figure 5). It is an important diagnosis to make as cochlear implantation is contraindicated. Conversely, the IAC, vestibule and semi-circular canals are normally sited; and CA has been further subgrouped based on the appearance of the latter two.^8^

(a) Cochlear aplasia with normal labyrinth.

This is frequently symmetrical, and the vestibule and SCCs are normal.

(b) Cochlear aplasia with a dilated vestibule (CADV)

This tends to be more asymmetric; both the SCCs and vestibule are variably dilated, and therefore appearances can mimic a common cavity (CC).^8^

Differentiating between the CADV and CC is clinically relevant as cochlear implantation is contraindicated in CA but potentially achievable in CC, depending on VIIIth nerve function. As a general rule, in CADV the IAC is normally sited, whereas in CC the IAC opens into its centre and may be more posteriorly directed, furthermore in CADV the cochlear promontory is not visualized.^8^

### Common cavity (CC)

**Figure 6.**
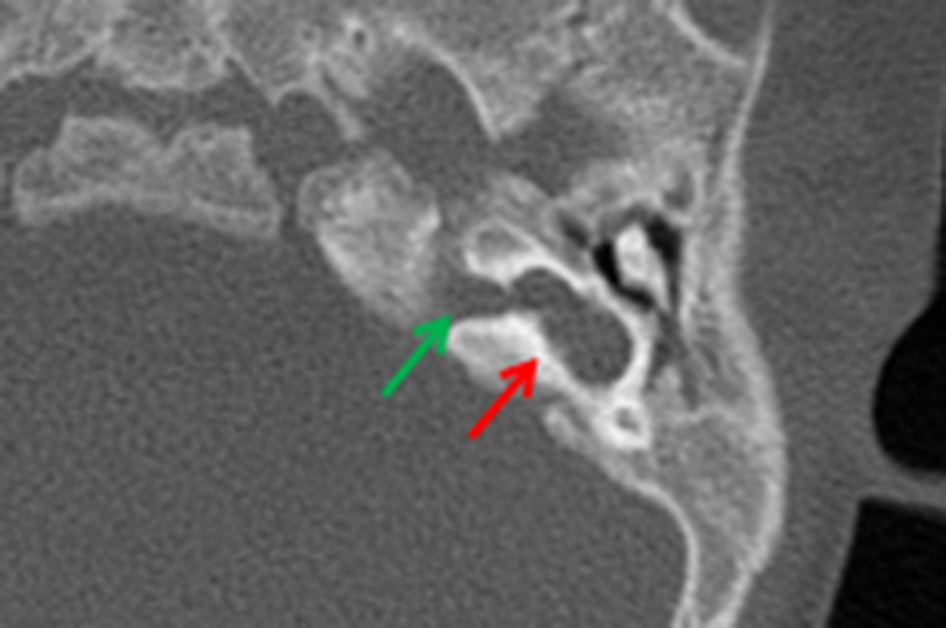
Showing typical common cavity without internal structure (red arrow). It takes its shape from the confluence of the outline of the cochlea and vestibule and is contiguous with the IAC (green arrow). Note that the cochlear promontory is not present given the absence of the basal turn of cochlea. IAC, internal auditory canal.

In a CC, the developmental arrest is thought to occur around the fourth gestational week^18^ with a resultant failure of cochlea and vestibule differentiation. Imaging demonstrates a cystic cavity formed by the confluence of their outline with no discernible internal architecture (Figure 6). Classically, the cavity is an ovoid or round shaped chamber which is wider (>10 mm average) than it is tall (>7 mm average).^1^ The SCCs may occasionally be normal but frequently are malformed. As mentioned above, the IAC frequently opens into the centre of the common cavity. Non-visualisation of the VIIIth nerve on 3 Tesla (3T) MRI may direct therapeutic options away from cochlear implantation and towards an auditory brainstem implantation (ABI).^8^

### Incomplete partitions (IP)

In this group of malformations, the cochlea and vestibule are clearly differentiated, and a key finding is normal (or near normal) sized external osseous structures. There are three subtypes of IP which are based on associated internal architecture anomalies, specifically the modiolus and interscalar septa.^8^ By definition all the IP are characterized by a normal sized cochlea^19^ and this allows radiological differentiation from some subtypes of cochlea hypoplasia.

### Type 1 incomplete partition (IP-1)

**Figure 7.**
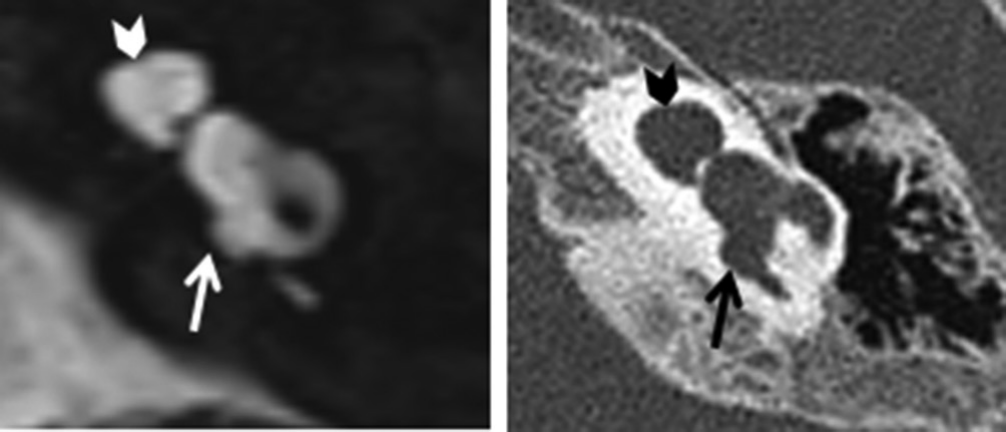
Type 1 incomplete partition appearances on MRI (left) and CT (right), the cochlea has normal dimension and no internal partitioning (arrowheads), the vestibule and semi-circular canals are dysmorphic (arrows).

This malformation has been termed “cystic cochleovestibular malformation” by Sennaroğlu et al^14^ and in contrast with the common cavity, the cochlea and vestibule can be differentiated from each other. The cochlea appears cystic with an aplastic/hypoplastic modiolus and interscalar septa^8^ (Figure 7). When combined with the frequently dilated vestibule a characteristic figure of eight appearance is often observed.^1^ The vestibular aqueduct is normal. All patients have an enlarged IAC, and many have deficiencies in the lamina cribrosa between the cochlea and IAC, and CSF may fill the cochlea.^8^ The enlarged IAC is important to identify pre-operatively as it increases both the risk of a perilymphatic gusher and meningitis post-surgery.^1^ The internal structure of the cochlea seems to be dependent, for its formation, on a sufficient blood supply during the embryological development. It has been proposed that impairment of vascularization (due to different causes) from the IAC may be responsible for IP-1.^3^

### Type II incomplete partition

**Figure 8.**
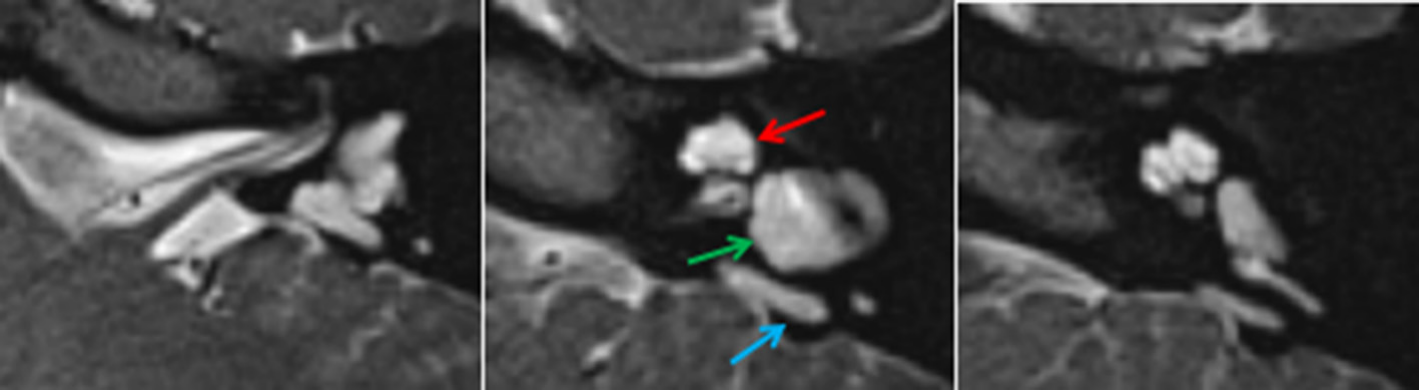
MRI appearances of incomplete partition Type 2, note cystic-like appearance of the upper cochlear turn with flattening of the interscalar septum laterally (red arrow), dilated and dysmorphic vestibule (green arrow) and enlarged endolymphatic sac (blue arrow).

This is the most common (>50%) of all cochlear malformations.^18^ There is coalescence of the middle and apical turns to form a cystic apex due to an apical defect in the interscalar septum (particularly in the lateral aspect of the cochlea) which is beautifully demonstrated on heavily *T*_2_W weighted, thin sliced, gradient echo MRI (Figure 8). The term “Mondini deformity” should be reserved for the triad observed by Carlo Mondini in 1791: this includes a cystic cochlear apex (but with a normal basal turn), a minimally dilated vestibule (with normal appearing semi-circular canals) and a dilated vestibular aqueduct containing an enlarged endolymphatic duct.^20^ The underlying pathophysiology is unclear. A proposed theory is that as a consequence of the dilated VA, CSF pulsation is transmitted back into the cochlea with a "third window" mechanism and the increased pressures result in a defective modiolus and dilated vestibule.^3^

### Type III IP

**Figure 9.**
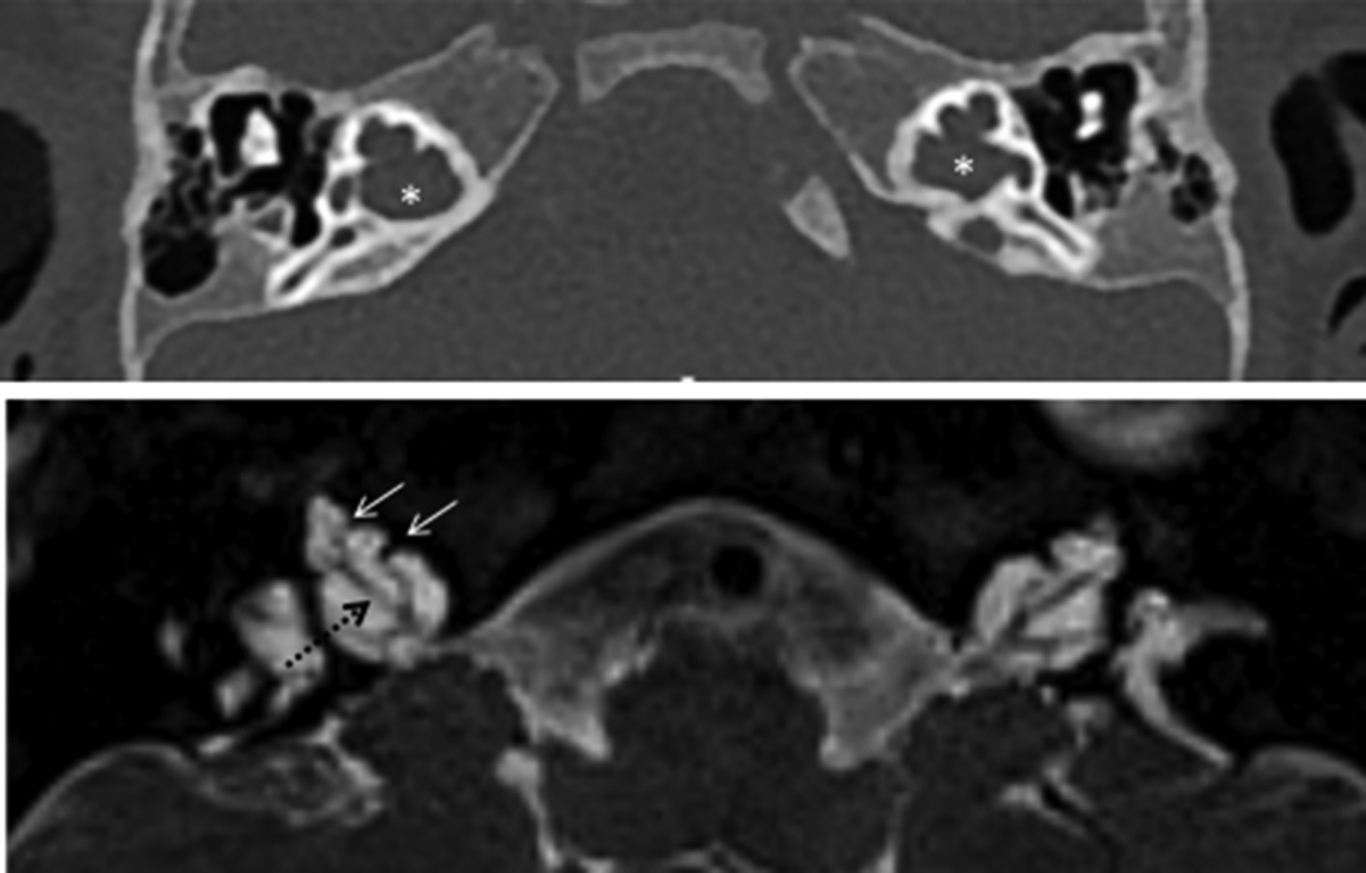
Typical CT (up) and MRI (down) appearances of incomplete partition Type 3. Note the enlarged internal auditory meatus (*), the presence of cochlear nerves (dotted arrow) and the preserved interscalar septi accounting for the typical shape of the cochlea in these patients (arrows).

In contrast, this is the rarest form of incomplete partitions and is characteristic of X linked deafness due to mutation in the POU3F4 gene.^21^ The interscalar septa are at least partially preserved but there is compete absence of the modiolus and no lamina cribrosa separating the cochlear base from a bulbous appearing IAC^21^(Figure 9). This abnormal connection between the peri-lymphatic system and the CSF places the patient at higher risk of a CSF leak^22^ and is sometimes known as an “X-linked stapes gusher.” The otic capsule surrounding the membranous labyrinth is thinner and appears to be formed by a thickening of the innermost endosteal layer without the normal enchondral and outer periosteal layers.^3^ The vestibular structures are normal (or the vestibular aqueduct may be slightly dilated) and the VIIth and VIIIth nerves are visible at MRI, although the labyrinthine segment of the VII is noted to course almost superiorly to the cochlea, rather than around it as normally seen on the axial slices.^8^

### Cochlear hypoplasia (CH)

As the name suggests, the cochlea is smaller than normal with four subtypes described according to internal architecture anomalies, namely of the modiolus, lamina spiralis and interscalar septa.^3^

1. CH-1

**Figure 10.**
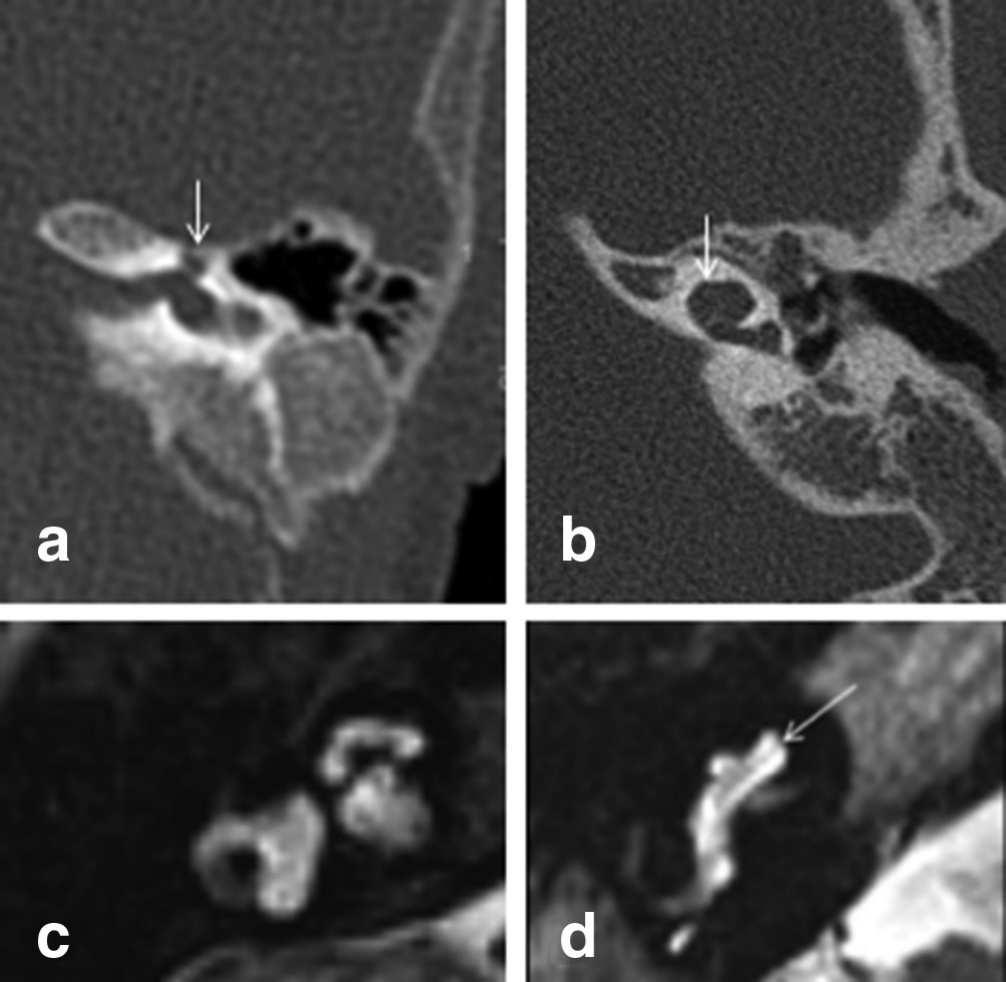
CT appearances of cochlear hypoplasia Type 1 (arrows in A) and Type 2 (arrow in B). Two different MRI appearances of cochlear hypoplasia Type 3 (C) and 4 (D) with relatively preserved internal structure and normal sized basal turn noted only in Type 4 (arrow in D).

This is the most severe form with a bud-like cochlea arising from the IAC and non-visualization of the modiolus and interscalar septa.^14^ Cochlear nerve absence or hypoplasia is usually observed^19^(Figure 10A).

2. CH-2

The cochlea is again small, but normal in outline with variable modiolus and interscalar septa (ISS) formation, almost looking like a small IP Type II (Figure 10B). The cochlear nerve and cochlear nerve canal can be normal.^8^ In cases where the modiolus is markedly underdeveloped, the connection with the IAC is wide and as a result the patient is at risk of electrode misplacement and post-surgical “CSF gushing”^8^

3. CH-3

Both the external outline and internal architecture are preserved; the cochlea is again smaller, with fewer turns and as such the anomaly is also known as a “cochlea with less than two turns”^8^ (Figure 10C). In reality, calculating the exact number of turns can be difficult due to its small size. The modiolus is also smaller and the interscalar septae shorter. Variable degrees of associated vestibular and SCCs hypoplasia are seen and the cochlear aperture is frequently hypoplastic/aplastic.^8^ This specific abnormality can be found (with different shape of the cochlea but always short size and relatively preserved internal structure) in syndromic causes of hearing loss such as SOX10 mutation or Branchio-Oto-Renal syndrome.^19^ More recently, a cochlear dysmorphology specific to Branchio-Oto-Renal has been described,^23^ whereby the apical and middle turns are anteriorly offset from a tapered basal turn; the so-called “unwound cochlea.”

4. CH-4

In contrast with CH-3, the basal turn is normal, and it is the hypoplastic middle and apical turns that contribute to the cochlea’s small size. The “cochlea with hypoplastic middle and apical turns”^8^ is usually bilateral and associated with an anteriorly dislocated labyrinthine segment of the VIIth nerve^24^ (Figure 10D).

### Dysplastic semi-circular canal

**Figure 11.**
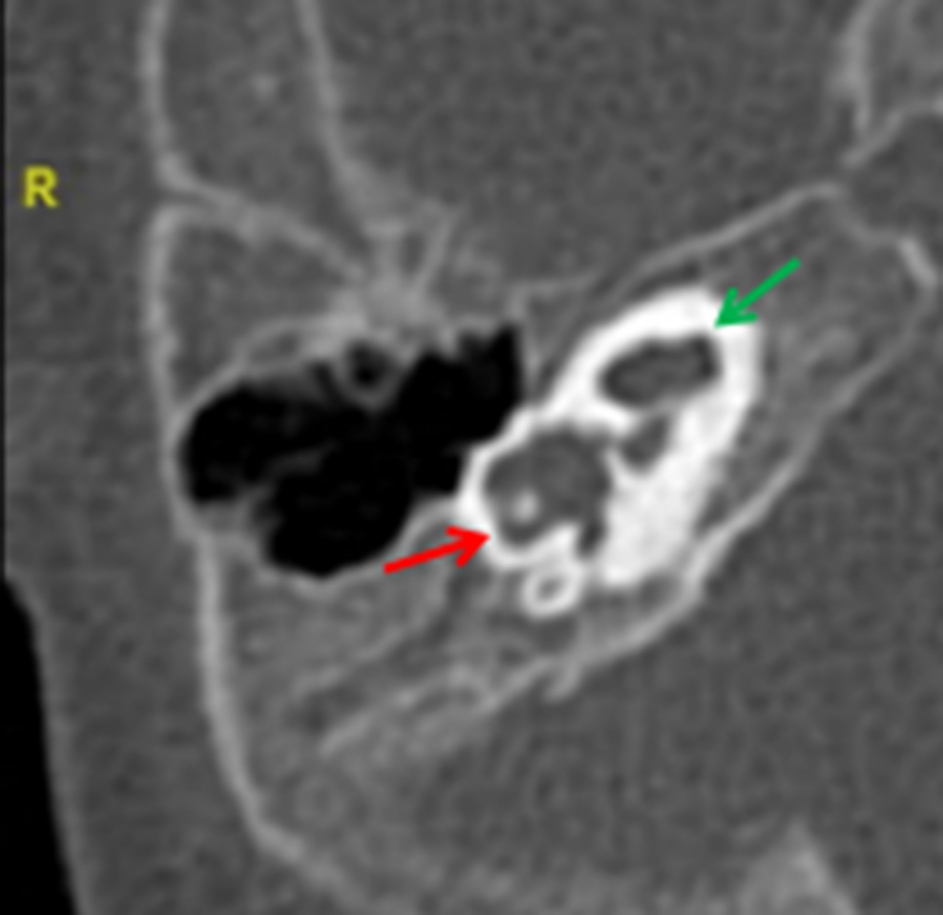
Dysplastic lateral semi-circular canal (red arrow), showing its typically short and wide morphology. The cochlea is abnormal with a hypoplastic interscalar septum between the middle and apical turn (green arrow).

The anomalous SCCs are characterized by a small bony island with typically short and wide canals. When the bony island is not formed it should be referred to as "persistent anlage of the SCC" (Figure 11). The lateral canal is last to form after the superior and posterior canals and therefore the lateral canal is almost always involved.^1^ Concomitant abnormalities of the cochlea may be seen depending on the embryological stage at which the arrest occurred^25^ Furthermore abnormal semi-circular canals in isolation or associated with other labyrinthine malformations are usually seen in the setting of various syndromes.^26^

### Absence or hypoplasia of the cochlear nerve

An abnormal stenotic or atretic IAC suggests an underlying cochlear nerve deformity but the two are not synonymous and therefore high resolution *T*_2_W MRI must always be performed for adequate cochlear nerve characterisation.

Three types of cochlear nerve deficiency/malformation have been described^27^

Type 1: The cochlear nerve is completely absent with a stenotic appearing IAC.

Type 2a: There is a common vestibulocochlear nerve but the cochlear nerve in the IAC is either aplastic or hypoplastic and there are associated inner ear anomalies.

Type 2b: The cochlear nerve is deficient, but the inner ear is otherwise normal.

Other associated anomalies are well seen on CT such as the “hypoplasia of the bony canal for the cochlear nerve.”^28^ This is a rare anomaly and refers to bony occlusion of the cochlear aperture, a tiny canal usually sited at the fundus of the IAC which normally houses the nerve as it passes to the cochlea.

### Enlarged vestibular aqueduct (EVA)

The finding of a dilated vestibular aqueduct (a bony structure and therefore best seen on CT) and endolymphatic duct and sac (only visible on MRI) is the most common radiological abnormality in patients with early onset SNHL and is bilateral in >90%.^24^ CT diagnosis of a dilated osseous VA is made when an axial measurement midway between the common crus and external aperture is >1 mm (Figure 8) or the measurement at the operculum is >2 mm.^7^ On thin section *T*_2_W MRI, the endolymphatic duct and sac are considered dilated when their diameter exceeds that of the ascending portion of the adjacent posterior SCC.^1^ Isolated enlarged vestibular aqueduct is rare and >80% will have an additional associated inner ear anomaly.^9^

## Conclusion

The early and accurate identification of structural inner ear anomalies that are amenable to surgery is central to the care of children with SNHL. Refinements in CT and MR techniques and an increasing awareness of the underlying pathophysiology enables better understanding of the specific type of defect, and ultimately facilitates a more informed discussion of the risks and benefits of cochlear implantation.
